# 

**Published:** 1895-05-11

**Authors:** 


"Johannis" THE KING OF NATURAL TABLE WATERS. "Johannis"

### THE HOSPITAL

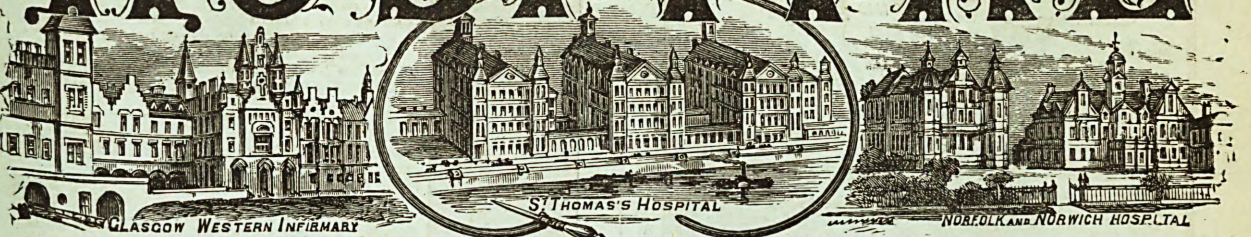

No. 450. Vol. XVIII.

Saturday, May 11th, 1895.

**WITH EXTRA NURSING SUPPLEMENT.**

[Registered at the General Post Office as a Newspaper for transmission in the United Kingdom and Abroad.]

**PRICE TWOPENCE.**

